# Supplementary material for: Individual housing of male C57BL/6J mice after weaning impairs growth and predisposes for obesity
Source: PLoS One. 2020 May 26;15(5):e0225488. doi: 10.1371/journal.pone.0225488 (PMC7250426; doi:10.1371/journal.pone.0225488)
Supplement: S1 Table — Data are means ± SEM; a = significant main effect of housing (p < 0.05); a = trend (0.05 < p <0.1); b = significant main effect of diet (p < 0.05); c = significant interaction housing x diet (p < 0.05); c = trend (0.05 < p <0.1); d = significantly different from IND-CTR (p < 0.05); e = significantly different from IND-WSD (p < 0.05); f = significantly different from SOC-CTR (p < 0.05). (DOCX) [file pone.0225488.s001.docx]

| Absolute body weight (g) at postnatal (PN) day | IND CTR  (*n=27*) | | | IND WSD  (*n=24*) | | | SOC CTR  (n*=22-23*) | | | SOC WSD  (*n=18-20*) | | | |  |  |
| --- | --- | --- | --- | --- | --- | --- | --- | --- | --- | --- | --- | --- | --- | --- | --- |
| PN42 | 24.11 | ± | 0.27 | 24.78 | ± | 0.30 | 26.24 | ± | 0.15^d,e^ | | 25.66 | ± | 0.36^d,e^ | a*,* c | |
| PN60 | 26.14 | ± | 0.32 | 30.25 | ± | 0.49 | 27.48 | ± | 0.30 | | 30.45 | ± | 0.69 | b, *c* | |
| PN80 | 28.96 | ± | 0.41^e^ | 35.88 | ± | 0.60^d^ | 29.04 | ± | 0.38^e^ | | 34.39 | ± | 1.92^d,f^ | a, b, c | |
| PN100 | 30.04 | ± | 0.46 | 37.78 | ± | 0.66 | 29.53 | ± | 0.40 | | 37.08 | ± | 1.06 | b | |
| PN126 | 30.14 | ± | 0.43 | 40.60 | ± | 0.83 | 29.07 | ± | 0.41 | | 39.12 | ± | 1.09 | *a*, b | |

**S1 Table. Absolute bodyweight of adult mice housed individually or socially and exposed to either control diet (CTR) or Western Style diet (WSD) between PN42 and PN126**.

Data are means ± SEM; ^a^ = significant main effect of housing (p < 0.05); *^a^* = trend (0.05 < p <0.1); ^b^ = significant main effect of diet (p < 0.05); ^c^ = significant interaction housing x diet (p < 0.05); *^c^* = trend (0.05 < p <0.1); ^d^ = significantly different from IND-CTR (p < 0.05); ^e^ = significantly different from IND-WSD (p < 0.05); ^f^ = significantly different from SOC-CTR (p < 0.05).
