# Supplementary material for: Individual housing of male C57BL/6J mice after weaning impairs growth and predisposes for obesity
Source: PLoS One. 2020 May 26;15(5):e0225488. doi: 10.1371/journal.pone.0225488 (PMC7250426; doi:10.1371/journal.pone.0225488)
Supplement: Attachment — Submitted filename: Comments Schipper.docx [file pone.0225488.s002.docx]

Authors have investigated metabolic performance and vulnerability to diet induced obesity in mice in response to individual or collective housing. Findings are clear and not over-interpreted. However novelty/originality is not clear and the paper seems to be incremental only. To address this point, I suggest inserting a mini-review based on previous papers in the introduction section, with a statement on what we know and what we do not know actually, just before giving the main objective of the present work.

Minor criticisms:

1) Please indicate issues from previous works on the same topic in the introduction section and state questions that still remain, which will improve interest for the present work. For instance studies that are already cited in the discussion should be included in this introduction, to reveal lacks or discordance on the effect of housing on energy homeostasis. Studies already cited in the discussion are:

Tsuduki et al. 2012;

Bibancos et al. 2007;

Lopez et al. 2015;

Shin et al 2018;

Nagy et al. 2002;

Moles et al. 2006.

Others studies not mentioned in the first version of this paper should appear here as well:

Breslin et al. Laboratory Animals 44, 2010;

Guo et al. Prog Neuropsychopharmacol Biol Psychiatry 28, 2004;

Nonogaki, Biochem Biophys Res Commun 378, 2009;

Nonogaki, Endocrinology 2007;

for instance…

3) More recent papers on thermoneutrality in mice and its impact on DIO models should be included. See Keijer et al. Mol Metab 25, 2019, and Fischer et al. Mol Metab 7, 2018, and related comments in 2019.

3) For statistical analysis, post hoc tests should be performed after ANOVA.
